# Viral Co-Infections in Pediatric Patients Hospitalized with Lower Tract Acute Respiratory Infections

**DOI:** 10.1371/journal.pone.0136526

**Published:** 2015-09-02

**Authors:** Miriam Cebey-López, Jethro Herberg, Jacobo Pardo-Seco, Alberto Gómez-Carballa, Nazareth Martinón-Torres, Antonio Salas, José María Martinón-Sánchez, Stuart Gormley, Edward Sumner, Colin Fink, Federico Martinón-Torres, GENDRES network

**Affiliations:** 1 Grupo de Investigación en Genética, Vacunas, Infecciones y Pediatría (GENVIP), Hospital Clínico Universitario and Universidade de Santiago de Compostela (USC), Galicia, Spain; 2 Section of Paediatrics, Division of Infectious Disease, Imperial College of London, South Kensington Campus, London, United Kingdom; 3 Unidade de Xenética, Departamento de Anatomía Patolóxica e Ciencias Forenses, and Instituto de Ciencias Forenses, Grupo de Medicina Xenómica (GMX), Facultade de Medicina, Universidade de Santiago de Compostela, Galicia, Spain; 4 Translational Pediatrics and Infectious Diseases Section, Department of Pediatrics, Hospital Clínico Universitario de Santiago, Santiago de Compostela, Galicia, Spain; 5 Micropathology Ltd., University of Warwick Science Park, Coventry, United Kingdom; Kliniken der Stadt Köln gGmbH, GERMANY

## Abstract

**Background:**

Molecular techniques can often reveal a broader range of pathogens in respiratory infections. We aim to investigate the prevalence and age pattern of viral co-infection in children hospitalized with lower tract acute respiratory infection (LT-ARI), using molecular techniques.

**Methods:**

A nested polymerase chain reaction approach was used to detect Influenza (A, B), metapneumovirus, respiratory syncytial virus (RSV), parainfluenza (1–4), rhinovirus, adenovirus (A—F), bocavirus and coronaviruses (NL63, 229E, OC43) in respiratory samples of children with acute respiratory infection prospectively admitted to any of the GENDRES network hospitals between 2011–2013. The results were corroborated in an independent cohort collected in the UK.

**Results:**

A total of 204 and 97 nasopharyngeal samples were collected in the GENDRES and UK cohorts, respectively. In both cohorts, RSV was the most frequent pathogen (52.9% and 36.1% of the cohorts, respectively). Co-infection with multiple viruses was found in 92 samples (45.1%) and 29 samples (29.9%), respectively; this was most frequent in the 12–24 months age group. The most frequently observed co-infection patterns were RSV—Rhinovirus (23 patients, 11.3%, GENDRES cohort) and RSV—bocavirus / bocavirus—influenza (5 patients, 5.2%, UK cohort).

**Conclusion:**

The presence of more than one virus in pediatric patients admitted to hospital with LT-ARI is very frequent and seems to peak at 12–24 months of age. The clinical significance of these findings is unclear but should warrant further analysis.

## Introduction

Lower tract acute respiratory infections (LT-ARI) are estimated to cause 75% of all acute illnesses and are the leading cause of hospitalization for infants and young children worldwide [1,2]. Most infections in children and adults are caused by viruses [1,3,4], including respiratory syncytial virus (RSV), influenza virus (IV), human parainfluenza virus (hPIV), adenovirus (AdV) and rhinovirus (hRV). In the past decade, several new respiratory viruses, including human metapneumovirus (hMPV) [5], new subtypes of human coronaviruses (hCoV) [1] and bocavirus (hBoV) [6], have been associated with LT-ARI, though their clinical importance requires clarification. The frequency of LT-ARI cases varies with age, among other factors.

The wider availability of molecular diagnosis techniques has allowed the identification of pathogens otherwise missed using conventional methods, and their use frequently leads to detection of more than one microorganism [7]. The importance of viral co-infections in the pathogenesis of LT-ARI is unclear; furthermore, the possible impact of age on viral co-infection prevalence is, to the best of our knowledge, unknown.

In the present study we aimed to estimate the prevalence of viral co-infection in children hospitalized with LT-ARI and to assess the impact of age in the rates of viral co-infection in these children.

## Material and Methods

### Study design and recruitment criteria

We conducted an observational, prospective study in Spain through a national hospital-based research network for pediatric respiratory research: GENDRES (Genetics, vitamin D and respiratory infections research network– www.gendres.org), which includes 13 Spanish tertiary hospitals. Eligible study participants were previously healthy children under 14 years of age admitted to a participating hospital with an LT-ARI diagnosis. LT-ARI was defined as any acute lower respiratory tract infection of sufficient severity to warrant admission to hospital. All types of LT-ARI were included, from bronchiolitis to pneumonia, with or without wheezing, fever, rhinorrhea or respiratory distress. A nasopharyngeal sample (aspirate/wash or swab) was obtained during admission in patients recruited between January 2011 and January 2013. A comparison cohort of 97 UK children admitted with LT-ARI, aged over 1 month, and sick enough to warrant blood tests, was recruited between 2009 and 2012 as part of the Immunopathology of Respiratory Infection Study (IRIS) at St Mary’s Hospital, London. These cohorts allowed for comparison of North and South European data from two regions with different climate and healthcare systems, as well as permitting validation of the Spanish findings on co-infection rate,and overall virus frequency. In both cohorts, recruited patients were admitted to PICU or ward. The Spanish cohort study was approved by the Ethical Committee of Clinical Investigation of Galicia (CEIC ref 2010/015) and by the regional ethics committees for each participating center, while the UK cohort study was approved by the St Mary’s Research Ethics Committee (REC 09/H0712/58). Written informed consent was obtained from a parent or legal guardian for each subject before study inclusion.

### Laboratory methods

Nasopharyngeal samples were obtained using a sterile feeding tube and injector for nasopharyngeal aspirate/wash or a sterile nylon swab without culture medium. Samples were kept at 4°C for up to 24 hours before storage at -80°C, pending transport on dry ice to the UK for virus detection by multiplex nested polymerase chain reaction (PCR) for 19 viruses: RSV; IV (A, B); HPIV types (1–4); AdV (A—F); hRV; HMPV; HCoV (NL63, 229E, OC43) and hBoV (gene targets and primer sources in Table 1). At the time of processing, viral material was eluted into a volume of 200 μl. Nucleic acid extracts were prepared using a QiagenMDx BioRobot. First round amplification was performed using 20 μl nucleic acid extract. Second round PCR was performed using 1 μl amplicon from first round PCR as template material. Reactions were run using a Lightcycler 480 with melt curve analysis for the detection of PCR products. Genotyping was performed by Sanger sequencing of second round PCR products when appropriate (AdV). Sequences were analysed using the BLAST algorithm.

**Table 1:** PCR gene targets and sources from which the primers were obtained.

Virus	Gene targets
Influenza A [8]	Gene N
Influenza B [9]	Gene M
Metapneumovirus [10]	Gene N
RSV [11]	Gene N
Parainfluenza 1 [8]	Gene HN
Parainfluenza 2 [12]	Gene HN
Parainfluenza 3 [12]	Gene HN
Parainfluenza 4 [13]	Gene P
Rhinovirus [14]	5’ UTR
Adenovirus (A-F) [15,16]	Hexon
Bocavirus [17]	NS encoding region
Coronavirus NL63 [18]	Gene N
Coronavirus 229E [19]	Gene M
Coronavirus OC43 [20]	Gene N

This multiplex nested PCR was performed in all samples independently of any diagnostic test carried out by the referring hospital.

### Data analysis

General data are shown as percentages or means with 95% confidence intervals (CI). Fisher’s exact test was used to study the association between the viruses and PICU admission. The level of statistical significance was set to 0.05. Statistical tests and Figures were carried out using R software v. 3.0.2 (R Core Team (2013). R: A language and environment for statistical computing. R Foundation for Statistical Computing, Vienna, Austria. ISBN 3-900051-07-0, URL http://www.R-project.org/http://www.r-project.org/).

## Results

### GENDRES cohort

The GENDRES cohort had a median age of 6.4 (first quartile: 2.2, third quartile:17.0) months and a male-to-female sex ratio of 1.7. One patient was excluded due to incomplete clinical data. The cohort included nasopharyngeal samples from 204 patients: 23 (11.3%) nasopharyngeal swabs and 181 (88.7%) nasopharyngeal aspirates/wash. No differences in findings were observed in relation to the method used for sample collection (data not shown).

**Fig 1:**
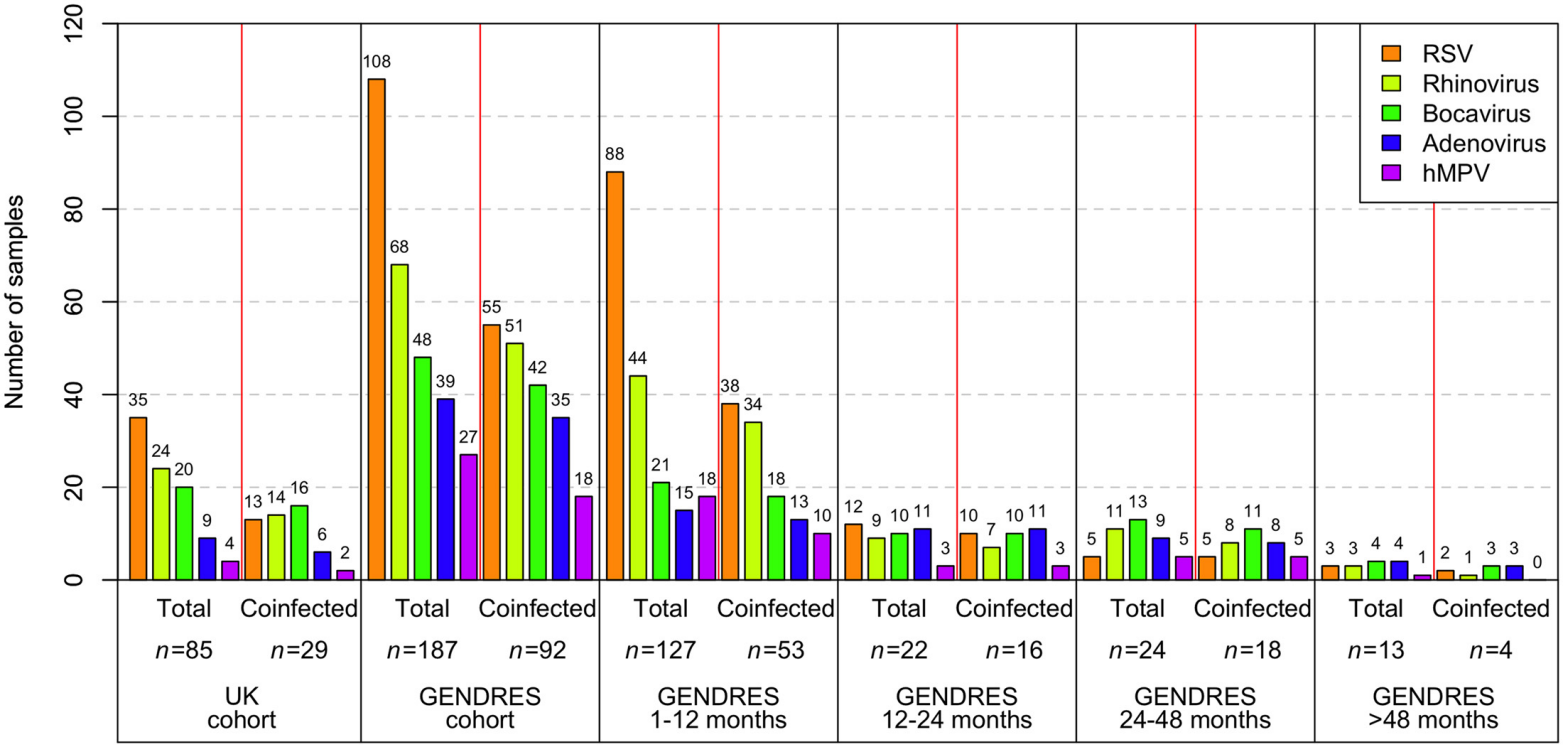
Pathogen prevalence in the main and replication cohorts shown as number detected in nasopharyngeal samples considering the age of the children. Only the more prevalent viruses are presented.

Molecular diagnostics identified at least one pathogen in 187 samples. Of these PCR positive samples, 73 had previously yielded negative results using conventional methodology—immunufluorescence assays and/or rapid techniques. Five samples (2.5%) were negative for both PCR and the initial diagnostic work-up. In 12 cases (5.9%) where the referring hospital had established a diagnosis, PCR was negative (RSV, *n* = 8; IV H1N1, *n* = 2; Mycoplasma, *n* = 1; and Influenza C, *n* = 1). By PCR multiplex assay, a single pathogen was detected in 95 (46.6%) children and two or more pathogens were detected in 92 (45.1%) patients, giving an overall detection rate of 91.7%. The most commonly detected virus was RSV (*n* = 108), followed by hRV (*n* = 68), hBoV (*n* = 48), AdV (*n* = 39), HMPV (*n* = 27), IV (*n* = 12), hPIV and hCoV (both *n* = 5) (Table 2). In co-infected samples, the most frequent combination of pathogens was RSV + hRV (*n* = 23) followed by RSV + hBoV (*n* = 10) and RSV + AdV (*n* = 7) (see Table 3). The virus most frequently found in dual infection was RSV (*n* = 42), followed by hRV (*n* = 35) (Table 2). RSV was observed with the same frequency as a single infection (*n* = 53) and as a co-infection agent (*n* = 55) (see Fig 1). However, hRV, IV, hBoV, AdV and hMPV were more frequently found in co-infections (Fig 1).

**Table 2:** Distribution of viral agents according to age in the GENDRES cohort (GEN) and UK cohort (UK). Data are presented as number of positive samples (percentage of evaluated samples) or the mean (standard deviation). Age is expressed in months.

	*Total n (%)*		*Single infection n (%)*		*Co-infection n (%)*		*Mean age (SD)*		*Age (months) [GEN n = 203; UK n = 97]*							
									*0–12*		*12–24*		*24–48*		*>48*	
	GEN	UK	GEN	UK	GEN	UK	GEN	UK	GEN	UK	GEN	UK	GEN	UK	GEN	UK
RSV	108	35	53	21	55	14	8.1	12.8	88	21	12	8	5	5	3	1
	(52.9)	(36.1)	(55.8)	(33.3)	(59.8)	(43.8)	(14.9)	(14.6)	(64.7)	(53.8)	(48.0)	(47.1)	(19.2)	(29.4)	(18.8)	(4.2)
Rhinovirus	68	24	17	11	51	13	14.4	40.9	44	7	9	5	11	6	3	6
	(33.3)	(24.7)	(17.9)	(17.5)	(55.4)	(40.6)	(17.4)	(46.3)	(32.4)	(17.9)	(36.0)	(29.4)	(42.3)	(35.3)	(18.8)	(25.0)
Bocavirus	48	19	6	3	42	16	22.5	31.9	21	4	10	8	13	4	4	3
	(23.5)	(19.6)	(6.3)	(4.8)	(45.7)	(50.0)	(27.4)	(35.8)	(15.4)	(10.3)	(40.0)	(47.1)	(50.0)	(23.5)	(25.0)	(12.5)
Adenovirus	39	9	4	3	35	6	21.4	51.2	15	2	11	1	9	3	4	3
	(19.1)	(9.3)	(4.2)	(4.8)	(38.0)	(18.8)	(20.1)	(48.9)	(11.0)	(5.1)	(44.0)	(5.9)	(34.6)	(17.6)	(25.0)	(12.5)
Metapneumovirus	27	4	9	2	18	2	16.8	48.5	18	1	3	1	5	1	1	1
	(13.2)	(4.1)	(9.5)	(3.2)	(19.6)	(6.3)	(27.7)	(57.2)	(13.2)	(2.6)	(12.0)	(5.9)	(19.2)	(5.9)	(6.3)	(4.2)
Influenza virus	12	23	4	13	8	10	35.7	45.9	2	5	4	5	4	5	2	8
	(5.9)	(23.7)	(4.2)	(20.6)	(8.7)	(31.3)	(43.2)	(44.0)	(1.5)	(12.8)	(16.0)	(29.4)	(15.4)	(29.4)	(12.5)	(33.3)
Parainfluenza	5	6	1	3	4	3	40.4	41.6	3	2	0	1	1	2	1	1
	(2.5)	(6.2)	(1.1)	(4.8)	(4.3)	(9.3)	(53.5)	(55.8)	(2.2)	(5.1)	(0.0)	(5.9)	(3.8)	(11.8)	(6.3)	(4.2)
Coronavirus	5	_	1	_	4	_	25.3	_	2	_	0	_	1	_	1	_
	(2.5)		(1.1)		(4.3)		(23.5)		(1.5)		(0.0)		(3.8)		(6.3)	
**Co-infection**	92	29	_	_	_	_	15.1	25.6	53	8	16	10	18	6	4	4
	(45.1)	(29.9)					(16.1)	(25.1)	(41.7)	(23.5)	(72.7)	(66.7)	(75.0)	(35.3)	(30.8)	(21.1)
**Total samples**	204	97	95	56	92	29	16.7	36.6	136	39	25	17	26	17	16	24
			(46.1)	(57.7)	(45.1)	(29.9)	(28.5)	(44.6)	(67.0)	(40.2)	(12.3)	(17.5)	(12.8)	(17.5)	(7.9)	(24.7)

**Table 3:** Associations among respiratory pathogens in hospitalized children in the GENDRES and UK cohorts.

	*GENDRES cohort*			*UK cohort*
*Pathogens detected*	*Number*	*% of positive cases*	*Number*	*% of positive cases*
**Co-infection, two pathogens**	65	34.2	24	28.2
RSV+hRV/hBov/AdV/MPV/IV	23/10/7/2/0	12.3/5.3/3.7/1.1/0.0	3/4/2/0/2	3.5/4.7/2.4/0.0/2.4
hRV+hBov/hCov/AdV/MPV/IV/HPIV	2/2/6/3/0/0	0.5/1.1/3.2/1.6/0.0/0.0	3/0/1/1/1/1	3.5/0.0/1.2/1.2/1.2/1.2
IV+hBov/MPV	2/1	1.1/0.5	5/0	5.9/0.0
hBov+AdV/hCov/MPV	1/1/4	0.5/0.5/2.1	0/0/0	0.0/0.0/0.0
AdV+HPIV/MPV	1/1	0.5/0.5	1/0	1.2/0.0
**Co-infection three pathogens**	21	11.3	4	4.7
RSV+hRV+AdV/hBov/hCov	3/3/1	1.6/1.6/0.5	0/0/0	0.0/0.0/0.0
RSV+hBov+IV/AdV	2/4	0.5/1.1	0/1	0.0/1.2
hRV+hBov+AdV/IV	4/0	2.1/0.0	1/1	1.2/1.2
IV+hBov+AdV	2	1.1	1	1.2
hBov+MPV+AdV/HPIV	2/2	1.1/1.1	0/0	0.0/0.0/
**Co-infection, four pathogens**	6	3.2	1	1.2
hRV+hBov+AdV+IV/MPV/HPIV	1/3/1	0.5/1.6/0.5	0	0.0
hRV+hBov+MPV+HPIV/RSV	1/0	0.5/0.0	0/1	0/1.2

### UK cohort

The UK cohort included samples from 97 patients, with a median age of 20.0 (first quartile: 7.0, third quartile: 48.7) months and a male-to-female ratio of 0.94. We identified at least one virus in 85 (87.6%) samples; in 12 samples no virus was identified. A single virus was present in 56 (57.7%) patients, and two or more viruses in 29 (29.9%) children. The most commonly detected virus was RSV (*n* = 35), followed by hRV (*n* = 24), IV (*n* = 23), hBoV (*n* = 19), AdV (*n* = 9), HMPV (*n* = 4) and hPIV (*n* = 6) (Table 2). In the co-infected samples, the most frequent combinations were RSV + hBoV and IV + hBoV (both *n* = 5). The viruses most frequently found in dual infections were RSV and hBoV (both *n* = 12), followed by hRV (*n* = 9) and IV (*n* = 8) (Table 3). hRV and IV were observed with similar frequencies in single infection and co-infection (hRV *n* = 11 vs 13; IV *n* = 13 vs 10), while hBoV and AdV were more frequently found as co-infections, and RSV was more commonly present as a single infection.

### Age patterns of infection

We compared co-infection frequencies according to age groups. In both cohorts, co-infection was found in all age groups. In the GENDRES cohort, there is a significant association between age and co-infection: in children aged 12–24 months (72.7% of infected patients; see Fig 1) and those aged 24–48 months (75.0% of infected patients; *P*-value = 0.001). In the UK cohort, there was also a significant association age and co-infection in patients aged 12–24 months (73.3% of the infected patients; *P*-value = 0.005).

In the GENDRES cohort, RSV infection affected younger children more frequently (mean age: 8.1 months; SD: 14.9) and hPIV was principally found in older patients (mean age: 40.4 months; SD: 53.5) (Table 2). In the UK cohort, RSV infection also affected younger children more frequently (mean age: 12.8 months; SD: 14.6) and AdV predominated in older patients (mean age: 51.2 months; SD: 48.9).

### PICU admission

There was no significant difference in the number of infections between the PICU and non-PICU cohorts. Although modest differences were found for hBoV (PICU: 15.8%—non PICU: 22.6%) and hMPV (PICU: 2.6%—non PICU: 14.0%) in the GENDRES cohort, these were not statistically significant. These differences were only observed in the UK cohort for hBoV (PICU: 16.3%—non PICU: 22.2%) (Table 4).

**Table 4:** Virus detection in PICU admitted patients in both cohorts. No differences were found when compared to those children not requiring PICU admission.

Virus	GENDRES cohort			UK cohort		
	PICU admission (*n* = 38) *n* (%)	No PICU admission (*n* = 93) *n* (%)	*P*-value	PICU admission (*n = 43) n* (%)	*No PICU admission* (*n = 54) n* (%)	*P*-value
**RSV**	24 (63.2)	56 (60.2)	0.844	17 (39.5)	18 (33.3)	0.671
**Rhinovirus**	15 (39.5)	28 (30.1)	0.312	11 (25.6)	13 (24.1)	1.000
**Bocavirus**	6 (15.8)	21 (22.6)	0.479	7 (16.3)	12 (22.2)	0.451
**Adenovirus**	5 (13.2)	14 (15.1)	1.000	3 (7.0)	6 (11.1)	0.727
**Metapneumovirus**	1 (2.6)	13 (14.0)	0.066	2 (4.7)	2 (3.7)	1.000
**Influenza**	1 (2.6)	4 (4.3)	1.000	8 (18.6)	15 (27.8)	0.342
**Parainfluenza**	1 (2.6)	3 (3.2)	1.000	1 (2.3)	5 (9.3)	0.223
**Coronavirus**	1 (2.6)	1 (1.1)	0.498	-	-	-

## Discussion

Multiple viruses are detected in at least one third of children hospitalized with LT-ARI. This rate reaches two thirds for patients in their second year of age.

With the introduction of molecular techniques, the detection of multiple co-infecting viruses has become common [21], though the prevalence of each virus varies between studies. Our results show that viral co-infection is frequent, particularly in children above one year of age: children aged 12–24 months had the highest number of detected viruses, which may reflect slower clearance (and perhaps increased pathogenicity) following primary infection by a virus, and an immature immune system [3,22]. We observed that co-infection rates were lower in older children in both cohorts, despite this group being prone to greater LT-ARI exposure through increased participation in shared childcare groups. This finding is inconsistent with two previous reports. In particular, Chorazy et al. [23] reported a non-significant increase in co-infection in children aged 6–12 months, and co-infection decreased after one year with increasing age. Peng et al. [24] reported that co-infection was more frequent in children between 3–6 years of age.

At least one respiratory pathogen was detected in 91.7% of the enrolled patients in the Spanish cohort and 87.2% in the UK cohort. This finding is in the upper end of the reported range in children (between 47% and 95%) [3,25,26]. Possible explanations for the wide differences in detection rates found in the literature include: (i) heterogeneity in studied populations (including genetic variability and predisposition), (ii) differences in respiratory symptoms at presentation (upper or lower respiratory symptoms), (iii) differences in the time of sampling, (iv) number of respiratory pathogens tested, and (v) the kind of diagnostic tests used [3,22,25]. Many patients had multiple respiratory viruses: 45.1% in the GENDRES cohort, and 29.9% in the UK cohort, which is again in the upper end of the reported range (17–41%) [3,27,28].

In 12 cases there was discordance between a negative PCR and a positive diagnostic pretest. This could be due to the different time of sampling, and/or it might be due to false positives, which are known to occur more frequently in rapid tests. We also found five negative samples (2.5%) for both PCR and pretest tested pathogens. These differences might be explained by the time and mode of collection of the samples. In some patients, the initial conventional viral test was performed on hospital admission samples, whilst PCR was performed using samples obtained after the patients were transferred to PICU and recruited for the study.

Some viruses were mainly present as co-infecting agents (hRV, IV, hBoV, AdV and hMPV) and rarely found as single pathogens. As previously reported [3,29,30], RSV was the most frequent pathogen in both cohorts, especially in younger children. The second virus most frequently detected by PCR was hRV. The clinical significance of a positive hRV PCR assay has been questioned, given that hRV has been detected in asymptomatic children even two weeks after the clinical symptoms had disappeared [31,32]. However, hRV has been identified as single pathogen in some ARIs in children [21]. In our study, hRV was found in one third of samples and as single pathogen in approximately 10% of the cases.

In the GENDRES cohort, infection by both RSV and hRV was the most common viral co-infection detected, but in the UK cohort the most common viral co-infections were RSV + hBoV and IV + hBoV. These differences most likely reflect the fact that UK patients were recruited during the 2009 pandemic influenza season, but they may also reflect local differences in epidemiology and recruitment (including a higher proportion of PICU cases in the UK cohort).

Bocavirus is a recently discovered virus that may cause ARIs, particularly in children, with the highest frequency found in hospitalized infants. Our results indicate that hBoV is commonly detected in respiratory samples of young children with LT-ARI, in agreement with previous reports [33,34]. In our study, hBoV was the third most frequently identified virus in the GENDRES cohort, after RSV and hRV, and the fourth in the UK cohort. Our detection rates in both the GENDRES and UK cohorts (23.5% and 19.6%, respectively) are higher than those in other published series, which have reported variable prevalence ranges of 1.5–19%. Methodological factors may explain these differences: our cohorts included only hospitalized children, whereas other studies included inpatients and outpatients [33,35]. hBoV was rarely found as a single infecting agent: in most cases (87.5%) it was found together with other respiratory viruses, as previously observed [34,36,37]. RSV, hRV, AdV and hMPV viruses were the most frequently observed co-pathogens, as observed by other authors [38–40].

In young children, hMPV is an important cause of bronchiolitis, accounting for 5–15% of all cases [4,41,42]. In our study we found 27 (13.2%) hMPV-positive samples in the GENDRES cohort. Of these, 66.7% of hMPV were detected as a co-infection with another respiratory virus, and 33.3% were found as a mono-infection. In the UK cohort four (4.1%) samples were hMPV-positive, including two with co-infection. Co-infection with hMPV has been proposed to increase disease severity in some studies [5,43,44], but not in others [45]. Dual infection with RSV is reportedly common, reflecting the overlapping seasonal distributions. One study reported that 70% of children with severe RSV bronchiolitis were co-infected with hMPV, suggesting that the disease caused by RSV may be augmented by a concurrent hMPV infection [44]. However, population-based and case control studies of hospitalized children have found that hMPV and RSV co-infections are uncommon [42,43]. In our study, the low proportion of mono-infected patients suggests that hMPV rarely produces clinically significant infection by itself, but co-infection of hMPV with RSV was also uncommon (only two cases).

Bezerra et al. [46] have observed that AdV is frequently detected as part of a co-infection, in contrast with the findings of Huang et al. [47]. AdV was reported to be responsible for 5–10% of ARI in children [11]. Our detection rate ranged between 9.3% (UK cohort) and 19.1% (GENDRES cohort) with a median age of 21.8 months.

Our study detected a broad range of common respiratory pathogens but it was not exhaustive, and indeed it may have missed as yet undescribed respiratory pathogens. The study considered only children admitted with LT-ARI, and did not include milder or asymptomatic infections. Several studies have shown that viruses can be found in children with no ARIs [22,48], and further research is needed to understand the respiratory viral carriage and infection. Although quantification of the virus load by PCR is possible, respiratory samples are heterogeneous, and different extractions of the same patient can lead to diverse results depending on chance variation in the amount of virus present in the aliquots extracted. Whilst the robustness of our findings is supported by the broad similarity between the two independent cohorts analyzed in the present study, its applicability to other populations is likely to be influenced by local epidemiological and host genetic factors.

In conclusion, the presence of more than one virus in children admitted to hospital with LT-AR ranged from one third to two thirds of these patients, depending on the age, and being particularly frequent in the second year of age. The co-infection pattern most frequently found was the combination of RSV and hRV. The clinical significance of co-infection remains difficult to establish from observational data. Further studies including also subjects with mild illness as well as healthy control groups are needed in order to better understand its clinical relevance.
